# Accelerated Weathering Testing (AWT) and Bacterial Biodegradation Effects on Poly(3-hydroxybutyrate-co-3-hydroxyvalerate) (PHBV)/Rapeseed Microfiber Biocomposites Properties

**DOI:** 10.3390/polym16050622

**Published:** 2024-02-24

**Authors:** Madara Žiganova, Remo Merijs-Meri, Jānis Zicāns, Agnese Ābele, Ivan Bochkov, Tatjana Ivanova

**Affiliations:** Institute of Chemistry and Chemistry Technology, Faculty of Natural Sciences and Technology, Riga Technical University, 3 Paula Valdena Street, LV-1048 Riga, Latvia; remo.merijs-meri@rtu.lv (R.M.-M.); janis.zicans@rtu.lv (J.Z.); agnese.abele_1@rtu.lv (A.Ā.); ivans.bockovs@rtu.lv (I.B.); tatjana.ivanova@rtu.lv (T.I.)

**Keywords:** rapeseed microfibers, alkali treatment, N-methylmorpholine N-oxide treatment, poly(3-hydroxybutyrate-co-3-hydroxyvalerate), biocomposite, biodegradation, accelerated weathering

## Abstract

In the context of sustainable materials, this study explores the effects of accelerated weathering testing and bacterial biodegradation on poly(3-hydroxybutyrate-co-3-hydroxyvalerate) (PHBV)/rapeseed microfiber biocomposites. Accelerated weathering, simulating outdoor environmental conditions, and bacterial biodegradation, representing natural degradation processes in soil, were employed to investigate the changes in the mechanical, thermal and morphological properties of these materials during its post-production life cycle. Attention was paid to the assessment of the change of structural, mechanical and calorimetric properties of alkali and N-methylmorpholine N-oxide (NMMO)-treated rapeseed microfiber (RS)-reinforced plasticized PHBV composites before and after accelerated weathering. Results revealed that accelerated weathering led to an increase in stiffness, but a reduction in tensile strength and elongation at break, of the investigated PHBV biocomposites. Additionally, during accelerated weathering, the crystallinity of PHBV biocomposites increased, especially in the presence of RS, due to both the hydrolytic degradation of the polymer matrix and the nucleating effect of the filler. It has been observed that an increase in PHBV crystallinity, determined by DSC measurements, correlates with the intensity ratio I_1225/1180_ obtained from FTIR-ATR data. The treatment of RS microfibers increased the biodegradation capability of the developed PHBV composites, especially in the case of chemically untreated RS. All the developed PHBV composites demonstrated faster biodegradation in comparison to neat PHBV matrix.

## 1. Introduction

Finite resources and climate issues require moving from a ‘take-make-dispose’ society to a carbon-neutral, environmentally sustainable, toxin-free and fully circular economy by 2050 [1]. Polyhydroxyalkanoate (PHA)-group polymers can be thermoplastically recycled, depolymerized to oligomers or monomers for use as renewable feedstock or converted to biomass by industrial or home composting [2]. Thus, PHA stands as a sustainable polymer for food packaging applications and beyond due to it nontoxicity, biocompatibility, recyclability and biodegradability. Each PHA molecule contains monomer units of certain (R)-hydroxy fatty acid connected each to other by ester bonds. However, applications of PHA often are limited due to it poor mechanical properties, such as low impact resistance, reduced elongation at break, and fragility. This is especially characteristic of the simplest member of the PHA group—polyhydroxy-3-butyrate (PHB). To overcome these issues to some extent, the length of the side chain may be increased. In general, PHAs may be categorized into different types on the basis of their structural chain length: short-chain-length PHAs, such as PHB, consist of monomeric building blocks of 3–5 carbons; medium-chain-length polymers, such as poly(3-hydroxyoctanoate), consist of 6–14 carbons; long-chain-length PHAs have monomeric building blocks with 15 or more carbon atoms, for example, poly(3-hydroxypentadecanoate) [3]. PHA copolymers also may be used instead of homopolymers. Building PHA structural chains from different monomeric units, in various proportions, can improve the properties of the material. One of the most studied PHB-based copolymers is polyhydroxy-3-butyrate-co-3-hydroxyvalerate (PHBV). An increase in the 3-hydroxyvalerate fraction in the copolymer reduces the melting point and significantly increases flexibility due to higher chain mobility [4]. PHA copolymers, especially at higher co-monomer content, however, possess a low modulus and thermal resistance. To improve stiffness, thermal resistance and dimension stability, polymers can be modified with anisodiametric particles as reinforcing phase. Modification of PHA with natural fibers is a good option for obtaining sustainable environmentally friendly materials for various applications without compromising its biodegradability [5]. Better properties of the composites usually are achieved by performing modification either of the fibers or the polymer matrix for increased interaction in the interfacial region. Furthermore, polymer reinforcement with low-cost fibers obtained from agricultural or forestry residues can reduce final costs of the materials. According to Xiaoying Zhao et al. [6], to reduce brittleness and costs, microfibers of invasive plants—hanarygrass (HG) and honeysuckle (HS)—may be used as reinforcing agents for PHBV at 5–20 wt % fiber content. The authors tested the interfacial bond shear strength (IBSS) of the PHBV composites containing untreated and treated microfibers. As expected, the IBSS for the untreated microfiber composites (1.9 ± 0.4 MPa; HS—2.3 ± 0.3 MPa) was smaller than that for the composite containing 2% NaOH-treated fibers (HG—3.3 ± 0.3 MPa; HS—2.7 ± 0.3 MPa). Berthet M. A. et al. [7] found that PHBV composite with wheat microfibers in the range 0–30 wt % possessed a significantly increased water vapor transmission rate (from 11 up to 110 g m^−2^ day^−1^); at the same time, Young’s modulus Young’s modulus was not significantly affected, which could fulfil the requirement for the packaging of respiring fresh food products, such as strawberries, thus enabling their preservation in a better way than currently used polyolefins. To compensate for the processability drop and decreased flexibility of composites, one of the most common approaches is plasticization. A broad range of PHA plasticizers have been examined, with citrate-based compounds as one of more effective ones. Thus, plasticization of PHB with triethyl citrate in the range 0–30 wt % led to an increase in the ultimate elongation from 5.8 to 6.9% [8]. MarieAlix Berthert et al. [9] compared wheat straw, brewers spent grains and olive mill particles’ potential use as fillers, with a mass content of 0–50% in PHBV for food-packaging applications. According to water vapor permeability results, PHBV composites with 20% wheat straw fibers demonstrated a 3.5-fold increment, being in consent with the requirements of respiring food products, whereas PHBV composites with 20% olive mills, demonstrating a 2.5-fold increment, could be more adapted for water-sensitive products. W. Frącz, G. Janowski, R. Smusz and M. Szumski [10] found that by adding up to 30% of various natural fibers with aspect ratio 10 to PHBV matrix, the tensile strength of the composite increased from 35 MPa to 42.9 MPa for hemp fibers and 40.18 MPa for flax fibers, but decreased to 30.68 MPa for wood fibers due to less regular distribution of the fibers within the matrix. Similar results were observed by Singh S. et. al. [11] for PHBV composites with wood a fiber content of 10–40 wt % at a somewhat lower aspect ratio (4–5). The tensile strength of this PHBV composite decreased from 21.42 MPa to 16.75 MPa when it was loaded with 40 wt % of wood fibers. The authors also found that tensile modulus of the investigated composite improved by 167% at the highest wood fiber content.

Usually, higher interaction, and hence properties, are achieved in the case of alkali-pretreated fibers [12,13]. Alkali pretreatment, however, requires the use of corrosive chemical compounds and creates large amount of alkaline wastewater. To overcome this issue, other fiber pretreatment methods, such as hydrothermal treatment, i.e., boiling in water [14,15] or steam explosion [16,17], may be used. In this aspect, N-methylmorpholine N-oxide (NMMO) may be considered as a non-toxic and biodegradable solvent for lignocellulosic fiber pre-treatment to reduce the amount of waxes and other undesirable impurities. Although NMMO has been used for wet spinning of Lyocel fibers, there is almost no evidence on the use of NMMO for lignocellulosic fiber pre-treatment aiming at improvement of interfacial interaction in the composites. 

Rosenau et al. [18] found that by modifying cellulostic fibers using an aqueous solution of NMMO in an “organic spinning process”, the mechanical properties of the obtained Lyocell fibers considerably improved, demonstrating very high dry and wet tensile strength, due to the formation of cellulose II. According to our previous research [19], NMMO treatment has a remarkable influence on the RS fiber chemical composition. It has been observed that the hemicellulose content in fibers decreased by 15% after NMMO treatment, thus providing better reinforcing capability.

Only a few research teams have investigated the influence of the composition of bio-based and biodegradable polymer composites with natural fibers on its weathering resistance and biodegradability. K.C. Batista et al. [20] found that with an increase in the peach palm particle (PPp) mass content from 10 to 25 wt %, the thermal stability of a PHBV composite decreased from 298 °C to 278 °C because of biodegradation in soil due to the increased hydrophylity of the composite. Micro-cavities, caused by the introduction of higher contents of PPp in the polymer matrix, allowed easier entering of water and microorganisms into the biocomposite matrix, resulting in enhanced degradation. In addition to that, plant residue could provide some nutrients such as polysaharides, proteins and minerals, which create favorable conditions for microorganisms [21].

However, to the authors’ knowledge, there is no sufficient evidence on the assessment of the change in the same PHBV composite’s properties with ligncellulosic fibers during accelerated aging in combination with biodegradation studies. Consequently, in the current research, attention is paid to the comparative evaluation of the effects of alkali and NMMO treatments of RS on the weathering resistance and biodegradability of PHBV/RS composites. It is expected that the study could provide more information on the performance of PHBV composites with lignocellulosic fibers during its life cycle and beyond.

## 2. Materials and Methods

### 2.1. Materials

Poly(3-hydroxybutyrate-co-3-hydroxyvalerate is a commercial product (PHBV, Ningbo City, China, TianAn Biopolymer: ENMAT Y1000) with 1 mol % HV 3-(hydroxyvalerate) content. Triethyl citrate (TEC, Burlington, MA, USA, Sigma Aldrich, Mw = 276.28 g·mol^−1^, ρ = 1.137 g·L^−1^) was used as a plasticizer. Winter rapeseed straw (RS) was collected as biomass waste from local farms Braslini, Pasiles and Susuri. RS were ground with a Retsch SM300 rotary grinder (Retsch GmbH, located in Haan, Germanya) a speed of 700 rpm using a 0.25 mm sieve. The obtained fibers were dried in an oven for 24 h at 60 °C and stored in closed plastic bags until further use. Sodium hydroxide pellets EMSURE were supplied by Sigma Aldrich (Sigma-Aldrich Chemie GmbH, located in Taufkirchen, Germany). A 50 wt % aqueous solution of NMMO was supplied by Merck (Biotecha Latvia, Riga, Latvia). Propyl gallate was purchased from Biosynth Carbosynth (Biosynth Carbosynth Ltd., located in Compton, Berkshire, United Kingdom).

### 2.2. Fiber Alkali Treatment (Mercerization)

According to our previous research [22], the most optimal conditions for treatment of RS were as follows. A certain amount (50 g) of the ground RS fibers was immersed in aqueous solution with a NaOH concentration of 2%, and the obtained suspension was mixed for 30 min. At the end of the mercerization, the fibers were washed several times using distilled water until neutral reaction. The treated fibers were dried in an oven at 60 °C for about 24 h and stored in closed plastic bags until further use.

### 2.3. Fiber NMMO Treatment

Firstly, the commercial NMMO solution (50 wt % in H_2_O) was concentrated to an 85% (*w*/*w*) solution by vacuum evaporation. Secondly, RS fibers were added to the prepared NMMO solution to achieve the optimal NMMO/water/cellulose ratio of 74:10:14. To prevent thermos-oxidative degradation, 1% (*w*/*w*) of propyl gallate was added. The vessel was placed in an oil bath at 90 °C under continuous mixing for different periods of time (1.3 h, 5 h and 30 h) to allow the treatment to occur. At the end of the treatment, the fibers were washed using distilled water until pH = 7. The treated fibers were dried in an oven at 60 °C for about 24 h and stored in closed plastic bags until further use.

### 2.4. Preperation of Composites

According to the previous research [23], the optimal amount of the TEC plasticizer was 20%, and the conditions for preparation of the composites with microfibers were as follows. The biopolymer and the microfibers were dried at 60 °C in a vacuum oven for 24 h. As shown in Table 1, systems with unmodified and two different types of modified RS microfibers were melt-compounded using a two-roll mill LRM-S-110/3E from Lab Tech Engineering Company Ltd., Phraeksa, Thailand. The mixing time was 3 min and the roll temperatures were 165 °C and 175 °C. Furthermore, the systems were milled at room temperature and 700 rpm using a Retsch cutting mill (SM300 Retsch GmbH, Haan, Germany) with a 6 mm sieve. The obtained flakes, with average dimensions of 3 mm × 2 mm, were used for manufacturing test specimens using compression molding as follows: 3 min pre-heating, 3 min pressing under 5 MPa pressure, 5 min cooling under pressure. Test specimens for mechanical property tests were cut from ~0.5 mm thick plates with dimensions of 60 mm × 100 mm obtained by hot pressing at 190 °C. Samples for bacterial biodegradation were cut from 1 mm thick plates with dimensions of 60 mm × 100 mm, similarly obtained by hot pressing.

### 2.5. Composite Characterization

#### 2.5.1. Weathering

A QUV accelerated weathering tester (Q-Lab Co., Westlake, OH, USA) equipped with fluorescent UVA-340 lamps (Q-Lab Co., USA) was used to simulate outdoor conditions in accordance with ISO 4892–3:2016 [24]. The test cycle consisted of ultraviolet irradiation (8 h) at black panel temperature 50 ± 3 °C and irradiance 0.75 W/m^2^, spray (15 min), and condensation (3 h and 45 min at 50 °C). A pre-determined amount of tensile test specimens was inserted into the QUV weathering chamber and exposed to the previously mentioned accelerated weathering conditions until 250 h and 500 h were reached.

#### 2.5.2. Fourier Transform Infrared Spectroscopy (FTIR)

FTIR spectra were obtained using a Thermo Fisher Scientific Nicolet 6700 spectrometer (Thermo Fisher Scientific, Waltham, Massachusetts, United States) via the attenuated total reflectance (ATR) technique. All spectra were recorded in the range of 650 to 4000 cm^−1^ with a resolution of 4 cm^−1^.

#### 2.5.3. Colorimetry

The color analysis of the prepared compositions was evaluated using a Ci7600 Sphere Benchtop Spectrophotometer (x-rite Pantone, Kentwood, MI, USA). Three parallel measurements were done using a total transmittance aperture of 6 mm, a wavelength range of 360–750 nm, a photometric resolution of 0.01%, and a white paper background due to the transparency of the samples. Lightness or luminance (L*) and chromaticity coordinates or base color parameters a* and b* (a*—the green-red component, b*—the blue-yellow component), saturation (C*) and hue angle (h°) were measured for ten replicate samples. The total color change (∆*E*) was calculated using Equation (1) according to ASTM D 2244-02 [25].

(1) $$∆E=\sqrt{∆L^{*2}+{∆a}^{*2}+{∆b}^{*2}}$$

where ∆L*, ∆a* and ∆b* are the differences between the initial and final values for L*, a* and b*, respectively.

#### 2.5.4. Differential Scanning Calorimetry (DSC)

Melting/crystallization behavior was evaluated using a Mettler/Toledo differential scanning calorimeter DSC 1/200W (Mettler-Toledo International Inc., Columbus, Ohio, United States). The specimen of approximately 10 mg was sealed in an aluminum pan and subjected to the following regime: the first heating run from 25 °C to 200 °C at a rate of 10 °C/min, ending with a holding at the target temperature for 5 min; the cooling run from 200 °C to −50 °C at a rate of 10 °C/min, ending with a holding at the target temperature for 5 min; and the second, final, heating run from −50 °C to 200 °C at a rate of 10 °C/min under a nitrogen atmosphere.

The degree of crystallinity (*χ*) was calculated using the following equation:

(2) $$\chi =\frac{\Delta H_{C}}{{\Delta H}_{m}^{{}^{\circ}}(1-w)}\times 100$$

where ∆*Hc* is the measured specific melting/crystallization enthalpy of PHBV phase and $∆H_{m}^{{}^{\circ}}$ is the melting enthalpy of the 100% crystalline PHB = 146 J/g [26].

#### 2.5.5. Tensile Properties

Tensile stress–strain characteristics were determined at a temperature of 20 °C in accordance with EN ISO 527 [27] using Zwick Roell material testing equipment, a BDO—FB020TN (Zwick Roell Group, Ulm, Germany), equipped with pneumatic grips. Type 5A test specimens were stretched at a constant deformation speed of 50 mm/min. Demonstrated values represent the averaged results of the measurements performed on 10 test specimens.

#### 2.5.6. Bacterial Biodegradation

Biodegradation at 58 ± 2 °C and 42% of soil humidity was carried out to characterize the biodegrabalility of the developed compositions by the rate of mass loss as a function of time. Compost soil (swamp peat) with a pH of 6.64 was acquired from a local distributor of UAB “Juknevičiaus Kompostas” (Vilnius, Lithuania). Similarly to Martins Nabels-Sneiders et al. [28], the samples were cut from a pressed plate with a thickness of 1300 μm. The samples were circular samples with d = 25.7 mm, and were encased between sieves and deposited in the soil at a depth of 1.5 cm using closed plastic containers. Samples were regularly recovered, dried in a vacuum oven at 60 °C for 4 h, weighed, photographed, and inserted back into the soil. The test was finished when the mass loss of the sample reached 50%.

## 3. Results and Discussion

### 3.1. Accelerated Weathering Impact on Properties

#### 3.1.1. Surface Chemistry (FTIR)

FTIR spectra in the absorption region of carbonyl bonds of unaged and aged PHBV and its composites with RS are shown in Figure S1a–i in Supplementary Materials. Liqing Wei et al. have previously reported that carbonyl absorption band intensities in the 1680–1800 cm^−1^ region may be related to the crystalline and amorphous parts in the polymer, i.e., absorption intensities of PHBV with 33 mol % of HV groups at 1720 cm^−1^ and 1740 cm^−1^ have been related to crystalline and amorphous parts in the polymer, allowing the calculation of the crystallinity index. In the case of the unirradiated PHBV with a low amount of HV units used in this research, intensities related to the crystalline and amorphous parts in the polymer may be observed at ca. 1718 cm^−1^ and ca. 1735 cm^−1^, respectively. The peak intensity ratio for unexposed PHBV was observed to be 3.4. After accelerated weathering, this ratio changed insignificantly. Kann et al. have minutely investigated the determination of crystallinity of PHA group polymers by means of FTIR, DSC and X-ray analysis [29]. The authors of this research have suggested the FTIR method for evaluation of the crystallinity of PHAs as a simpler and easier method in comparison to X-ray diffraction or scattering. They have also found correlations between FTIR results and DSC measurements. In their contribution, they suggested to determine the crystallinity index of PHAs between absorption bands 1230 cm^−1^ and 1184–1186 cm^−1^, related to the conformational band at helical chains, observed only in the crystalline phase and C-O-C asymmetric stretching, pronounced in the amorphous phase, respectively. In the current research, the respective absorption bands for crystalline and amorphous parts of PHBV have been observed at ca. 1220–1230 cm^−1^ and 1180 cm^−1^ (Figure S2a–i in Supplementary Materials). By calculating the crystallinity ratio from these absorption bands (see Table 2), it may be observed that by increasing the exposure time, the crystallinity of PHBV is increased, which confirms with findings from DSC measurements (Section 3.1.3). By evaluating the effect of weathering time, it may also be observed that the intensity of the small peak at ca 1685 cm^−1^ increased with increasing weathering time, which is most probably attributed to partial hydrolysis of PHBV and the formation of carboxylic acids during the aging process. It may also be observed that the chosen method of the fiber surface treatment does not considerably affect the position of the carbonyl peak.

Another common indicator of aging of polymers is the carbonyl index, which was calculated from the FTIR spectra by comparing the main carbonyl absorbance peak at 1718 cm^−1^ with the peak relatively stable under the conditions of accelerated weathering, such as the peak at 1379 cm^−1^, which is attributable to –CH_3_ group fluctuations. It is observed that I_1718/1379_ for all the investigated composites decreases with increasing weathering time. Similar results have been obtained by Ana Antuanes et al. [30], who have observed degradation in both the crystalline and amorphous phases of PHBV nanocomposites with TiO_2_. It is expected that decrease in I_1718/1379_ occurs because of hydrolytic degradation of PHBV during accelerated weathering. Iggui et al. [31] have demonstrated that thedecrease in the carbonyl index because of hydrolytic aging is clearly related to the reduced molecular weight of PHBV.

#### 3.1.2. Colorimetry

**Figure 1:**
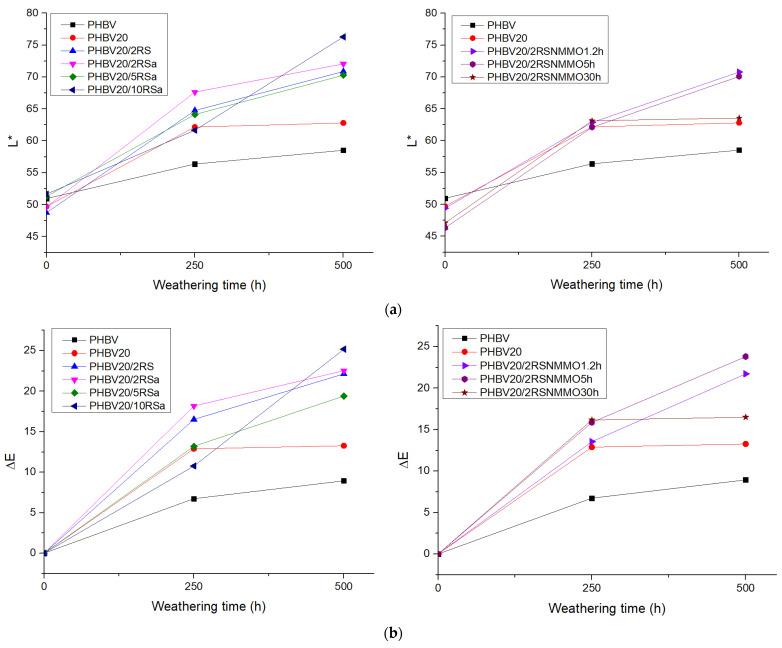
Change of colorimetric parameters L* (**a**) and Δ*E* (**b**) with accelerated weathering time.

Examination of the color fastness of the PHBV and its composites led to obtaining the photometric images shown in Table 3, whereas the colorimetric parameters are summarized in Figure 1 and Supplementary Materials Table S1. As one can see, the fibers’ treatment with alkali or modification with TEC didn’t considerably change lightness (L* changes not more than 2 units in comparison to that of neat polymer matrix) of the developed PHBV composites. The effect of NMMO treatment is greater; however, it may be connected with longer treatment times, which were 3 to 60 times longer in comparison to those used during mercerization. In general, NMMO-treated fibers yielded a darker surface color of PHBV composites, especially if treated for longer time. As a result of UV irradiation and increased moisture during accelerated weathering, the surface of the PHBV composite samples became rougher, most probably because of partial thermal and hydrolytic degradation of the biopolymer matrix. In addition, all the investigated systems during aging tended to fade out, as shown by increasing L* values. This is characteristic for natural fiber-reinforced biopolymer composites, as may be confirmed by results obtained by Aneta Tor-Świątek et al. [32] for linen fiber–biopolymer composites or A.T. Michel for hemp fiber-reinforced PHB composites. As may be expected, a larger color change Δ*E* was observed for the investigated PHBV composites with higher amounts of microfibers.

#### 3.1.3. Thermal Properties (DSC)

**Figure 2:**
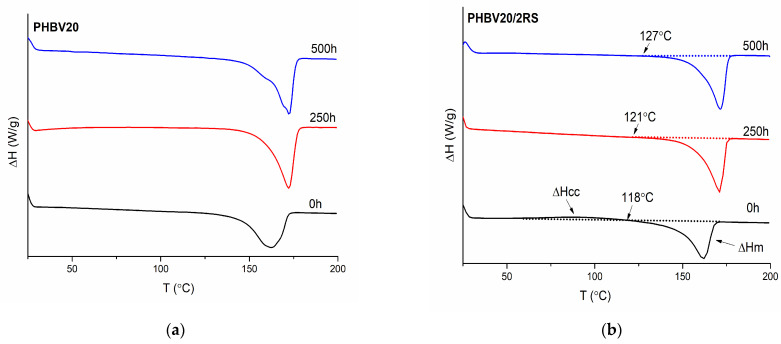
DSC thermograms change of PHBV20 (**a**) and PHBV20/2RS (**b**) during accelerated weathering process.

**Figure 3:**
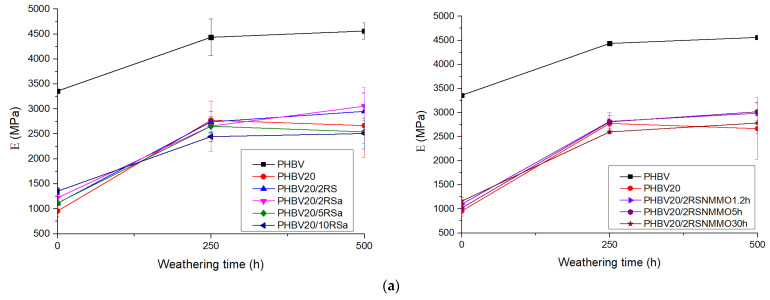

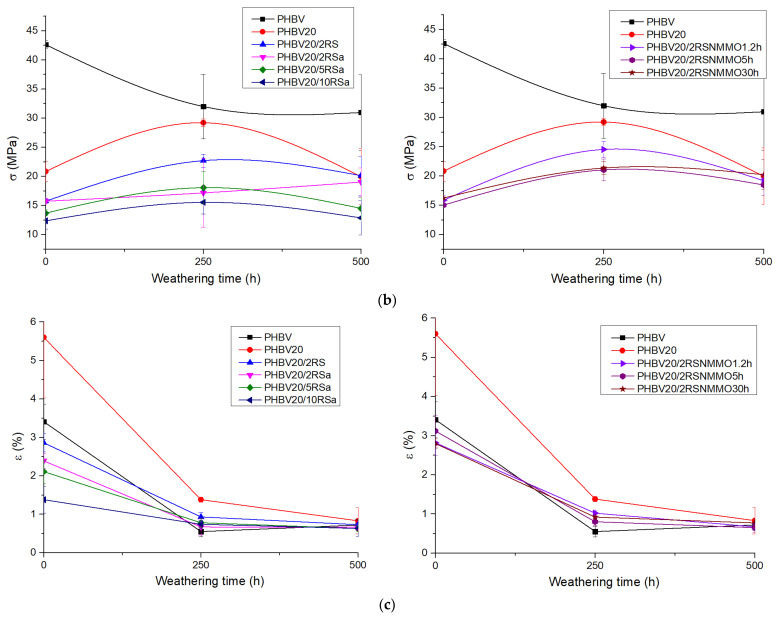
Young’s modulus E (**a**), stress at break σ_B_ (**b**), and ultimate deformation ε_B_ (**c**), of the PHBV and PHBV biocomposites before accelerated weathering and after 250 h and 500 h of the combined exposure of UV irradiation, temperature, water sprinkling and condensation.

DSC analysis was used to determine changes in the melting/crystallization behavior of PHBV and its plasticized composites before and after accelerated weathering. The results of DSC analysis are summarized in Figure 2 and Table 4. From the first-run DSC thermograms of plasticized PHBV and its composite with RS, depicted in Figure 2, it is possible to determine the cold-crystallization and melting regions of the PHBV crystalline phase. Glass transition relaxation of the PHBV phase is hardly detectable from DSC measurements and is therefore not analyzed. It should be mentioned, however, that the glass transition temperature of PHBV and its composites usually fluctuates within a range of 0–27 °C, depending on the measurement method [23,33]. As demonstrated in Figure 3, as soon as RS is added, cold crystallization of the PHBV phase may be observed. This is most probably related to the effect of RS on the crystallization behavior of PHBV from melt during compression moulding of the test specimens. After exposure to 250 h of accelerated weathering, the cold crystallization region of PHBV + 2RS composite was lost, which may be explained by increased temperature during the test run (60 °C during the UV irradiation cycle and 50 °C during the condensation cycle). This may result in a more complete structural arrangement of the PHBV phase. In addition, because of accelerated weathering, the melting peak of the PHBV crystalline phase is shifted towards higher temperatures by ca. 10 °C. This may be due to structural rearrangement of the PHBV phase within both the plasticized matrix without and with RS. Further increment of exposure time does not considerably change the maximum temperature of the PHBV melting peak. However, the shape of the plasticized PHBV melting peak after 500 h of accelerated aging is changed due to certain degradation of the PHBV phase with the formation of a greater variety of crystalline structures (please see Figure S3 in Supplementary Materials). First-run DSC thermograms give an important information about the investigated materials. A more detailed layout of the calorimetric data from the first-run DSC experiments is given in Table 5, where, besides relaxation peak maximum temperatures, corresponding onset and offset temperatures are given, accompanied by respective enthalpy values, from which values of crystallinity degree have been calculated. Cold crystallization peak enthalpy was also considered when calculating the crystallinity degree of the investigated systems. However, cold crystallization resulted in a broad flat peak with unexpressed maximum, allowing the assumption that cold crystallization temperature changed insignificantly with addition of the rapeseed microfibers. The maximum melting temperature also changed to a relatively small extent, i.e., within 6 °C. Comparatively larger changes of *T_m_* have been observed for the composites, containing NMMO-modified fibers with the highest treatment time. In general, by increasing the fibers’ content, some increase in the onset, maximum and offset temperatures of the melting is observed, resulting in the shift of the melting peaks towards somewhat higher temperatures. The total crystallinity degree of the plasticized PHBV phase, however, is decreased for the composites with fibers. Similarly to PHBV and its composite with untreated rapeseed fibers, after 250 h of exposure, there is an increment in the melting peak temperatures of the composites containing treated fibers. By further increasing exposure time up to 500 h, the changes in these calorimetric parameters are comparatively smaller, denoting that the greatest structural changes have occurred already, prior to the exposure time of 250 h. However, it should be mentioned that first-run DSC thermograms are considerably influenced by thermal history events; therefore, more reliable data, characterizing structural changes of the investigated PHBV composites upon aging, may be found from cooling or second-run DSC thermograms, which are also displayed in Table 5. From the cooling thermograms’ data, only one exothermic peak may be observed, which is related to crystallization of the PHBV phase in the composites. As shown, the crystallization temperature maximum is not considerably changed along with the addition of rapeseed microfibers, fluctuating within the range of 7 °C. There are also no considerable differences in crystallization onset and offset temperatures, but crystallization occurs in a somewhat narrower temperature range for rapeseed fiber–containing composites, which may be attributed to promotion of crystallization by the fibers. After 250 h of exposure, crystallization from the melt begins at somewhat higher temperatures, whereas it is more pronounced for the composites, especially in the case of higher RSa fiber content and longer NMMO treatment times of the RS. A similar trend is also observed after 500 h of irradiation, although there is practically no change in the beginning of the crystallization from the melt in comparison to systems exposed for 250 h. Disregarding this, after accelerated weathering, a considerable increase in the crystallinity degree of the composites with fibers is observed, which may be explained by partial hydrolytic degradation of the polymer matrix, resulting in easier crystallizing of macromolecular fragments. It may be concluded that RS microfibers tend to act as nucleants, promoting crystallization of the polymer matrix, especially in the case of PHBV composites. In [30,31], it has been also reported that cellulose particles in a PHBV matrix act like nucleating agent, inducing PHBV to crystallize at lower temperatures because of the reduced energy barrier. Table 5 also contains results from the second DSC run after controlled cooling from the melt. It may be observed that in the second DSC run thermograms, no cold crystallization peaks are observed, most probably because the cooling rate was enough to ensure complete crystallization of PHBV. Consequently, all second heating run DSC thermograms display two melting peaks, the first of which is attributed to melting of less ordered crystallites, formed during controlled cooling. In general, it may be observed that the melting range of the investigated systems after the controlled cooling is shifted to somewhat lower temperatures. In addition, PHBV melting peak temperatures almost do not change with increasing fiber content. More pronounced effects are observed after exposing the investigated compositions to accelerated aging. It may be observed clearly that a shift of the PHBV melting peak towards higher temperatures occurs. In addition, the evolvement of at least three different crystalline structures may be observed along with increasing exposure time. For example, in the case of plasticized PHBV, the lowest melting maxima, observed at ca 155 °C is related to less ordered crystalline structures, whereas its intensity evidently increases with exposure time. The second temperature maxima, observed dominantly for unexposed PHBV is at 165 °C. After exposure for 250 h this temperature maxima is decreased and new temperature maxima at 171 °C appears. Further increment of exposure time up to 500 h results in decrease of this temperature maxima to 168 °C. Similar trends have been observed also in the case of RS fibers containing composites. There is a trend of increased intensity of the highest temperature maxima around 171 °C with increasing both, the RSa fiber content and the RS treatment time with NMMO. This may be related to the fact that larger amount of fibers or more fibrillated fibers more efficiently promote crystallization of PHBV matrix, especially in the case of partially degraded macromolecular fragments at the highest exposure time. As a result crystallinity degree of PHBV has been considerably increased with increasing both RSa fiber content and treatment time of RS fibers by NMMO.

#### 3.1.4. Tensile Properties

The tensile properties of the investigated PHBV composites before and after accelerated weathering are summarized in Figure 3, allowing the assessment of the mechanical property changes of the material during its expected life cycle. As shown in Figure 3a, the tensile modulus E of all the investigated systems increases by increasing the accelerated weathering time. In general, the highest E values, independently from the exposure time, have been observed for neat PHBV. It is not surprising as, by the addition of 20% of TEC as the plasticizer, the stiffness of all the investigated unexposed composites is decreased. If the fibers are added, the modulus of the unexposed plasticized PHBV composites is increased from 956 MPa to 1354 MPa. The highest E value is observed for the composite containing 10% of alkali-treated RS. Larger modulus increment is hindered by the fact that no chemical interaction occurs between the hydrophobic polymer matrix and the hydrophilic fibrous reinforcement, as demonstrated also in the FTIR measurements. By increasing the accelerated weathering time up to 500 h, the E of the plasticized PHBV and its composites increases up to two times, whereas the increase in the ultimate strength of the investigated systems is limited to 250 h. In addition, the reinforcing effect of RS fibers is practically lost after 250 h of accelerated aging. However, after 500 h of accelerated aging, the PHBV composites with 2 wt % of the filler demonstrate ca. 10% higher E values in comparison to the neat biopolymer matrix. Evidently, this is related to the higher crystallinity of PHBV in the composite, as demonstrated by DSC measurements. Unfortunately, the observed increase in E, by increasing the accelerated weathering time, is accompanied with increased brittleness, resulting in rupture of the composites at low ultimate elongation values, even lower than 1.5%. This is due to the limited outdoor exploitation time of the developed composites, where the material is subjected to elevated external temperature, direct water exposure and condensation. This does not exclude short-term application of the material indoors or for a limited time, even in outdoor conditions.

### 3.2. Biodegradation in Soil

Disregarding the material properties for the expected application, its life cycle is limited. The great advantage of the investigated PHBV composites are their biodegradability, as they totally consist of natural and bio-based constituents. Consequently, in this section, the biodegradability of the developed composites is addressed. Biodegradation is influenced by many factors, such as microbial activity, soil moisture content, temperature, the pH of the environment, the exposed surface area and the composition and molecular weight of the polymer and its crystallinity [34,35]. Rapeseed fibers primarily consist of cellulose, a hydrophilic substance that readily allows water and microorganisms to permeate through its structure to attack the encapsulating polymer matrix, not only via the external environment but also internally. In Table 5, the biodegradation process of the developed PHBV composites in soil is pictured, revealing the evolution of the biodegradation process, accompanied with gradual surface roughening and the development of pits, grooves, cavities and other surface defects, resulting in bulk structure disintegration. 

It is shown that biodegradation starts in the PHBV matrix, predominantly in the amorphous phase, revealing the RS fibers, which is especially evident for the PHBV20/10RSa composite. However, except for the plasticized PHBV composite with 2% of untreated RS, biodegradation does not result in fragmentation of the test specimens along with the test run. In the case of the plasticized PHBV composite with untreated RS, fragmentation occurs within 13 days. Disregarding the composition, the investigated test specimens demonstrated ongoing color pattern change during biodegradation in soil. In Table 5, the evolvement of spotted uneven surface images of the test specimens is demonstrated clearly, as biodegradation time is increased.

**Figure 4:**
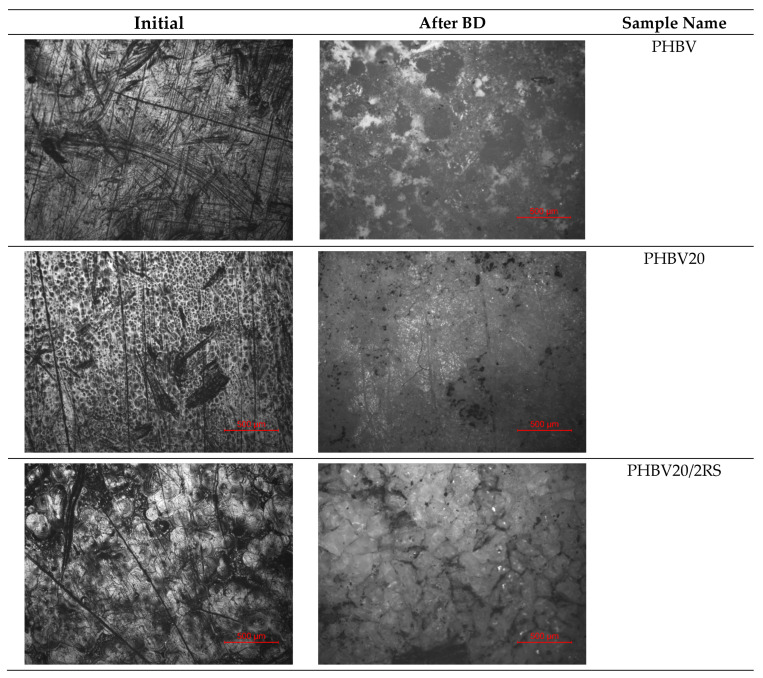

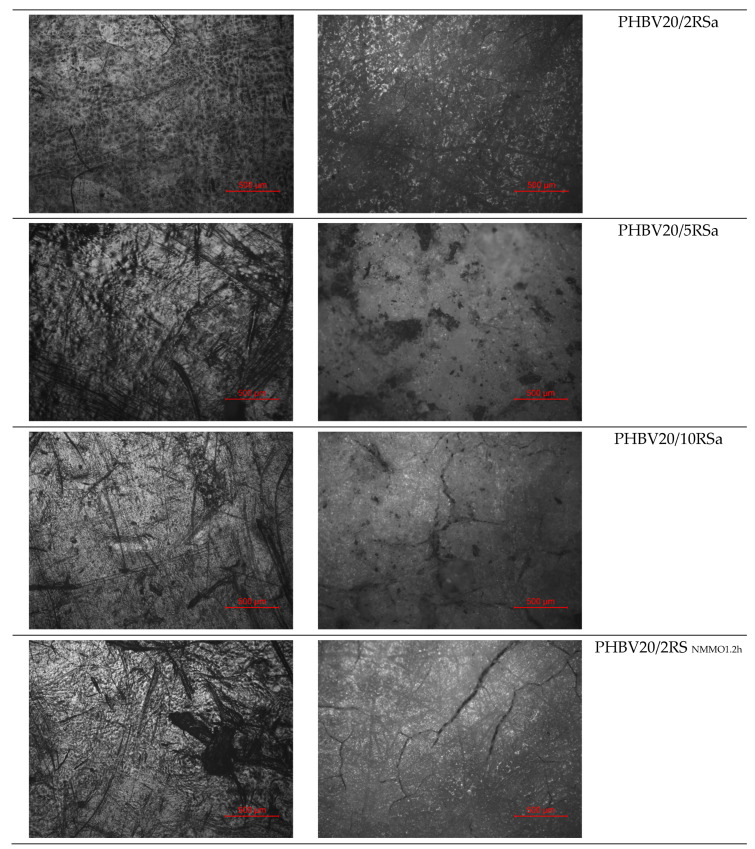

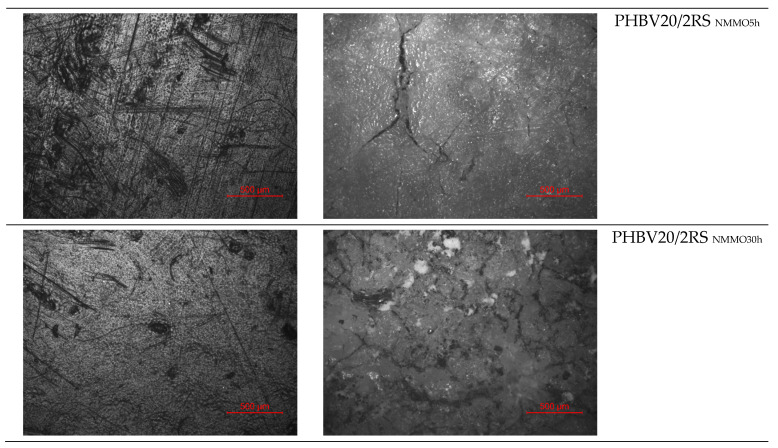
Optical microscopy pictures of PHBV and its plasticized composite samples before and after 3 month biodegradation.

In Figure 4, optical microscopy images of the investigated compositions are summarized before and after the biodegradation test. It is evident that after the biodegradation test, images become more blurry, which indirectly testifies to the increased surface roughness of the test specimens. The surface of the plasticized PHBV + 2%RS test specimens demonstrate a clearly visible evenly spread multi-fracture network across the surface of the test specimen, testifying to considerable fragmentation. For RSa-containing plasticized PHBV composites surface images do not reveal the development of considerable fractures, except of the composition with the highest RSa content, which may be due to the fact that fracturing initially occurs in the PHBV and RS interface. In the case of NMMO-treated RS-containing composites, fracturing is more visible in comparison to RSa-reinforced PHBV composites, whereas fracturing increases with the increase in NMMO treatment time.

**Figure 5:**
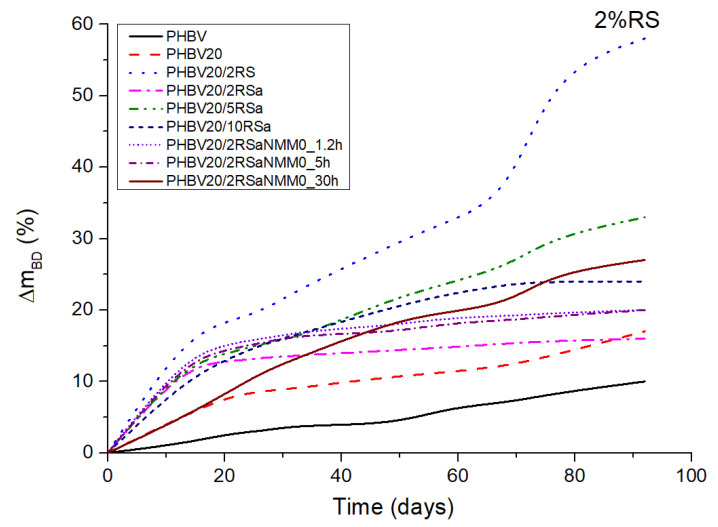
Mass loss during degradation of PHBV and its plasticized composites.

In Figure 5, the mass loss kinetics of the investigated PHBV compositions are shown. It is evident that all the investigated composites demonstrate faster biodegradability in comparison to the neat polymer matrix. This is likely because of the increased hydrophilicity of the composites with the introduction of the fibers in the polymer matrix. TEC also contributes to degradation of PHBV by promoting its hydrolytic degradation. The fastest biodegradation was observed for the PHBV composition with 2 wt % of untreated RS. Surface erosion of the test specimens may have influenced the shape of the biodegradation curves of other PHBV compositions. The reason for faster biodegradation of the composite with untreated RS fibers is most probably lower interfacial adhesion between the polymer matrix and the fiber. Contrarily, due to the fiber treatment, part of the hemicelluloses and lignin are removed along with other impurities, increasing the purified fibers’ interaction with the polymer matrix, and hence making biodegradation more difficult. It has been previously reported that fiber treatment influences its structure because of fibrillation and removal of impurities for improved adhesion with polymer [6].

## 4. Conclusions

This research focuses on the assessment of the structural, mechanical and calorimetric properties change of alkali- and NMMO-treated TEC-plasticized PHBV composites during accelerated weathering and biodegradation in soil. The main results of the study allow the following conclusions. Accelerated weathering during 500 h of the combined exposure of UV irradiation, increased temperature, water sprinkling and condensation led to an increase in stiffness, but a reduction in tensile strength and elongation at break, of the investigated plasticized PHBV biocomposites. It was determined that the largest change in these indicators of mechanical properties occurred prior to 250 h of the exposure, when the reinforcing effect of RS fibers on the modulus of the composites has been lost. The main reasons for the decreased properties were attributed to hydrolytic degradation of PHBV as well as thermal and photochemical destruction of the composites, especially in the presence of RS fibers. Changes in the mechanical properties with increased exposure time were accompanied by increased crystallinity of PHBV, especially in the presence of RS, which was believed to promote crystallization as a nucleant, in addition to the effect of partially degraded PHBV. DSC results correlated with the increment of the crystallinity index, determined as a FTIR-ATR peak intensity ratio between vibrations in crystalline and amorphous-phase I_1225/1180_. Destruction of the PHBV matrix during accelerated weathering was also confirmed by the appearance of multipeak behavior in DSC scans of the UV-exposed composites. In respect to biodegradation, it was observed that all the developed PHBV composites demonstrated faster biodegradation in comparison to neat PHBV matrix. It was suggested that biodegradation primarily occurred in RS-concentrated areas, especially for chemically untreated RS fibers. Contrarily, treatment of RS microfibers delayed biodegradation of the developed PHBV composites due to increased adhesion between the polymer matrix and RS fibers because of partial removal of waxes, lignin and hemicelluloses during the surface treatment.

## Figures and Tables

**Table 1:** Compositions of the obtained composite systems.

Code	PHBV (wt %)	RS (wt %)	RSa (wt %)	RS_NMMO_ (h)	TEC (wt %)
PHBV	100	−	−	−	0
PHBV20	80	−	−	−	20
PHBV20/2RS	78	2	−	−	20
PHBV20/2RSa	78	−	2	−	20
PHBV20/5RSa	75	−	5	−	20
PHBV20/10RSa	70	−	10	−	20
PHBV20/2RS_NMMO1.2h_	78	−	−	1 h 20 min	20
PHBV20/2RS_NMMO5h_	78	−	−	5 h	20
PHBV20/2RS_NMMO30h_	78	−	−	30 h	20

RS—rapeseed straw microfibers; RSa—alkali treated rapeseed straw microfibers; RS_NMMO_—rapeseed straw microfibers treated with NMMO, TEC—triethyl citrate.

**Table 2:** Carbonyl index and crystalline/amorphous ratios for PHBV and the PHBV biocomposites, both before and after weathering.

Code	Carbonyl Index I_1718/1379_	Crystallinity Index I_1718/1735_	Crystallinity Index I_1225/1180_
PHBV 0 h	3.3	3.4	1.3
PHBV 250 h	3.3	3.1	1.2
PHBV 500 h	3.2	3.1	1.2
PHBV20 0 h	3.8	3.3	1.0
PHBV20 250 h	3.6	3.4	1.3
PHBV20 500 h	3.2	3.2	1.2
PHBV20/2RS 0 h	3.5	2.6	0.9
PHBV20/2RS 250 h	3.0	3.2	1.2
PHBV20/2RS 500 h	3.0	3.1	1.2
PHBV20/2RSa 0 h	4.2	3.0	0.8
PHBV20/2RSa 250 h	3.1	3.2	1.2
PHBV20/2RSa 500 h	2.6	2.9	1.1
PHBV20/5RSa 0 h	4.3	3.1	0.8
PHBV20/5RSa 250 h	3.0	3.2	1.1
PHBV20/5RSa 500 h	3.0	3.2	1.2
PHBV20/10RSa 0 h	4.0	2.9	0.9
PHBV20/10RSa 250 h	3.3	3.3	1.2
PHBV20/10RSa 500 h	3.1	3.2	1.1
PHBV20/2RS_NMMO1.2h_ 0 h	4.2	3.0	0.8
PHBV20/2RS_NMMO1.2h_ 250 h	3.3	3.0	1.2
PHBV20/2RS_NMMO1.2h_ 500 h	3.4	3.5	1.2
PHBV20/2RS_NMMO5h_ 0 h	4.3	2.9	0.8
PHBV20/2RS_NMMO5h_ 250 h	3.0	3.3	1.1
PHBV20/2RS_NMMO5h_ 500 h	3.0	2.9	1.2
PHBV + 20/2RS_NMMO30h_ 0 h	3.9	3.7	0.8
PHBV20/2RS_NMMO30h_ 250 h	3.4	3.4	1.2
PHBV20/2RS_NMMO30h_ 500 h	2.4	2.6	1.1

**Table 3:** Photometric images of color change of PHBV and its plasticized composites during accelerated weathering for 0 h, 250 h, 500 h.

Sample Code	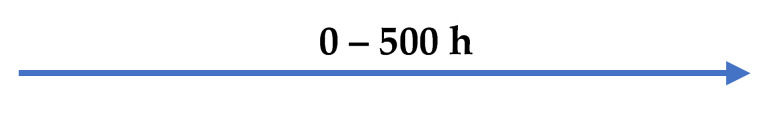
PHBV	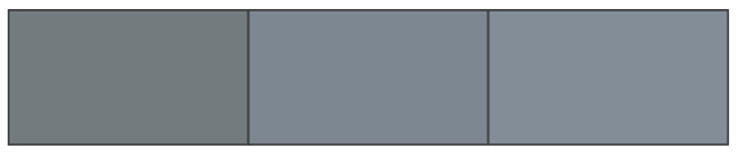
PHBV20	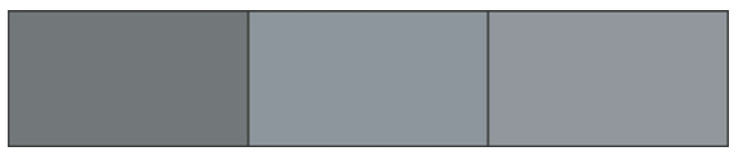
PHBV20/2RS	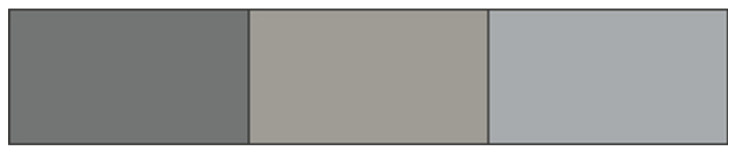
PHBV20/2RSa	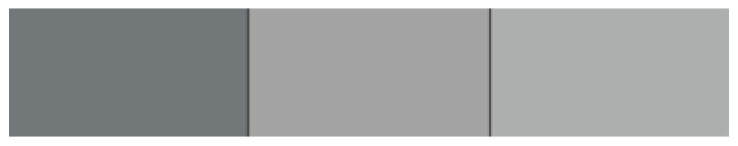
PHBV20/5RSa	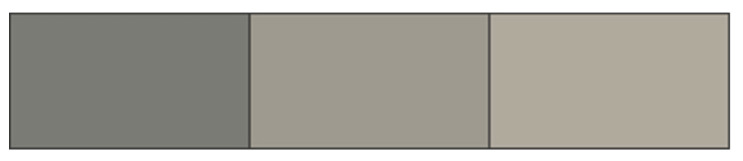
PHBV20/10RSa	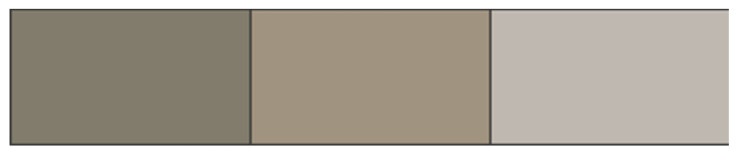
PHBV20/2RS_NMMO1.2h_	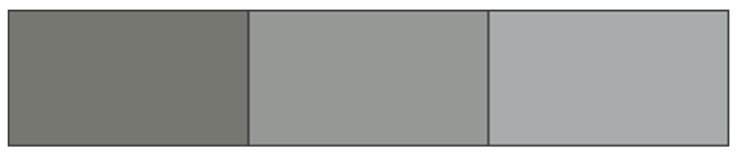
PHBV20/2RS_NMMO5h_	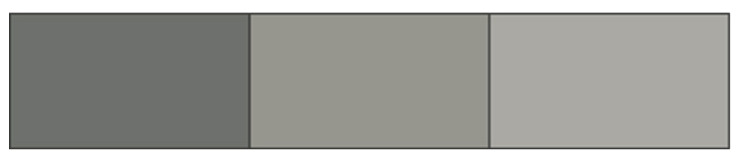
PHBV20/2RS_NMMO30h_	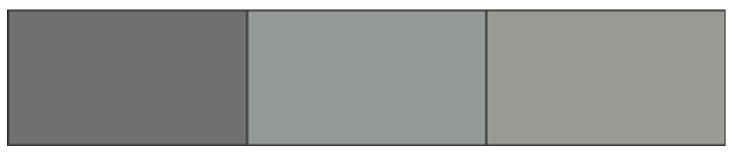

**Table 4:** Change of calorimetric properties of PHBV and the plasticized PHBV composites during accelerated weathering process (first heating run).

	Calorimetric Parameter– Aging Time	T_cc_												T_m_												χ, %											
Sample Code		0 h				250 h				500 h				0 h				250 h				500 h				0 h				250 h				500 h			
PHBV		-				-				-				175				175				172				64				55				55			
PHBV20		-				-				-				163				172				173				57				63				63			
PHBV20/2RS		90				-				-				162				171				171				54				65				71			
PHBV20/2RSa		91				-				-				162				172				171				55				68				67			
PHBV20/5RSa		91				-				-				167				175				172				47				70				66			
PHBV20/10RSa		91				-				-				165				172				172				47				70				75			
PHBV20/2RS_NMMO1.2h_		99				-				-				165				171				173				47				69				69			
PHBV20/2RS_NMMO5h_		102				-				-				164				170				174				42				68				70			
PHBV20/2RS_NMMO30h_		104				-				-				168				176				172				52				67				69			
(Cooling)																																					
	**Calorimetric Parameter–** **Aging Time**	**T_m_**																		**χ, %**																	
**Sample** **Code**		**0 h**						**250 h**						**500 h**						**0 h**						**250 h**						**500 h**					
PHBV		83						89						91						47						46						38					
PHBV20		75						82						73						44						50						47					
PHBV20/2RS		76						85						82						45						54						59					
PHBV20/2RSa		74						87						77						45						56						56					
PHBV20/5RSa		74						91						88						44						58						59					
PHBV20/10RSa		77						92						92						47						61						63					
PHBV20/2RS_NMMO1.2h_		73						88						82						44						77						59					
PHBV20/2RS_NMMO5h_		71						86						77						41						55						57					
PHBV20/2RS_NMMO30h_		70						89						81						44						56						59					
(second heating run)																																					
	**Calorimetric Parameter–Aging Time**	**T_cc_**									**T_m1_**									**T_m2_**									**χ, %**								
**Sample** **Code**		**0 h**			**250 h**			**500 h**			**0 h**			**250 h**			**500 h**			**0 h**			**250 h**			**500 h**			**0 h**			**250 h**			**500 h**		
PHBV		95			103			99			167			168			166			172			174			172			56			48			49		
PHBV20		91			92			91			154			164			156			165			172			168			58			57			52		
PHBV20/2RS		90			97			96			154			163			162			165			171			171			54			52			58		
PHBV20/2RSa		91			97			90			155			162			160			166			171			170			55			57			57		
PHBV20/5RSa		94			100			97			154			166			164			165			172			171			44			62			58		
PHBV20/10RSa		95			97			100			155			165			165			165			171			171			49			66			62		
PHBV20/2RS_NMMO1.2h_		90			97			94			154			164			163			165			171			170			53			67			64		
PHBV20/2RS_NMMO5h_		91			101			91			153			163			161			165			171			170			49			55			59		
PHBV20/2RS_NMMO30h_		94			94			93			154			165			162			165			171			170			50			65			65		

**Table 5:** Photos of PHBV and PHBV composites during the biodegradation process in the composting conditions.

Sample Code	Time (Days)									
	0	13	21	27	33	49	58	69	76	92
PHBV	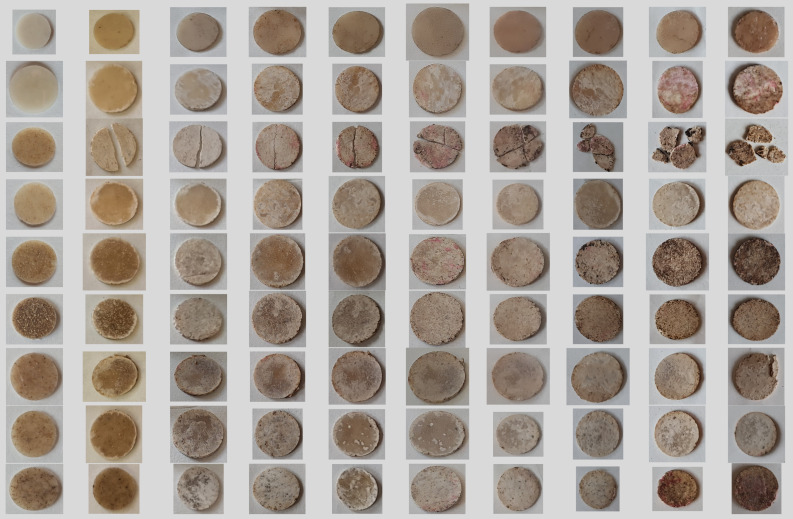									
PHBV20										
PHBV20/2RS										
PHBV20/2RSa										
PHBV20/5RSa										
PHBV20/10RSa										
PHBV20/2RS_NMM0_1.2h_										
PHBV20/2RS_NMM0_5h_										
PHBV20/2RS_NMM0_30h_										

## Data Availability

Data are contained within the article and Supplementary Materials.

## Supplementary Materials

The following supporting information can be downloaded at: https://www.mdpi.com/article/10.3390/polym16050622/s1, Figure S1: FT-IR spectra at ester carbonyl bond range (1800–1680 cm^−1^) change during weathering time for neat PHBV (a) and all obtained composites (b–i) according to presented legends; Figure S2: FT-IR spectra at range (1300–1000 cm−1) change during weathering time for neat PHBV (a) and all obtained composites (b–i) according to presented legends; Figure S3: DSC thermograms; Table S1: Colorimetric parameters of PHBV and its plasticized composites during accelerated weathering for 0 h, 250 h, 500 h.
