# Supplementary material for: Accelerated Weathering Testing (AWT) and Bacterial Biodegradation Effects on Poly(3-hydroxybutyrate-co-3-hydroxyvalerate) (PHBV)/Rapeseed Microfiber Biocomposites Properties
Source: Polymers (Basel). 2024 Feb 24;16(5):622. doi: 10.3390/polym16050622 (PMC10934408; doi:10.3390/polym16050622)
Supplement: Supplementary file 1 [file polymers-16-00622-s001.zip › Table S1.pdf]

Table S1. Colorimetric parameters of PHBV and its plasticized composites during accelerated weathering for 0 h, 250 h, 500 h.

Sample name	L*	a*	b*	C*	h°	ΔE
**PHBV 0 h**	51	-3	-3	4	225	0
**PHBV 250 h**	56	-2	-7	7	251	7
**PHBV 500 h**	59	-2	-7	8	254	9
**PHBV20 0 h**	50	-2	-1	3	208	0
**PHBV20 250 h**	62	-2	-5	5	246	13
**PHBV20 500 h**	63	-1	-3	4	246	13
**PHBV20/2RS 0 h**	49	-1	0	1	181	0
**PHBV20/2RS 250 h**	65	0	4	4	94	17
**PHBV20/2RS 500 h**	71	-1	-1	2	233	22
**PHBV20/2RSa 0 h**	50	-2	-2	3	223	0
**PHBV20/2RSa 250 h**	68	-1	0	1	153	18
**PHBV20/2RSa 500 h**	72	0	0	0	198	23
**PHBV20/5RSa 0 h**	51	-2	3	4	114	0
**PHBV20/5RSa 250 h**	64	0	6	6	88	13
**PHBV20/5RSa 500 h**	70	1	7	7	82	19
**PHBV20/10RSa 0 h**	52	-1	10	10	94	0
**PHBV20/10RSa 250 h**	62	3	12	12	78	11
**PHBV20/10RSa 500 h**	76	1	5	5	74	25
**PHBV20/2RS$_{NMMO1.2h}$ 0 h**	49	-1	3	3	107	0
**PHBV20/2RS$_{NMMO1.2h}$ 250 h**	63	-2	2	2	132	14
**PHBV20/2RS$_{NMMO1.2h}$ 500 h**	71	-1	-1	1	216	22
**PHBV20/2RS$_{NMMO5h}$ 0 h**	46	-2	2	3	129	0
**PHBV20/2RS$_{NMMO5h}$ 250 h**	62	-1	4	4	105	16
**PHBV20/2RS$_{NMMO5h}$ 500 h**	70	0	3	3	100	24
**PHBV+20/2RS$_{NMMO30h}$ 0 h**	47	-2	2	2	132	0
**PHBV20/2RS$_{NMMO30h}$ 250 h**	63	-1	0	1	191	16
**PHBV20/2RS$_{NMMO30h}$ 500 h**	64	-1	2	2	109	16
